# Knowledge, attitudes, and practices of liver cirrhosis patients regarding dietary nutrition

**DOI:** 10.3389/fnut.2025.1707256

**Published:** 2026-01-06

**Authors:** Yu Feng, Yuqian Li, Pengyan Wu, Xiaoxiao Qian, Baofang Zhang, Futang Li

**Affiliations:** 1 Department of Clinical Nutrition, The Affiliated Hospital of Guizhou Medical University, Guiyang, China; 2 Infection Department, The Affiliated Hospital of Guizhou Medical University, Guiyang, China; 3 Department of Hepatobiliary Surgery, The Affiliated Hospital of Guizhou Medical University, Guiyang, China

**Keywords:** cross-sectional study, Diet-food-and nutrition, health knowledge-attitudes-practice, liver cirrhosis, nutritional status, patient education, risk factor

## Abstract

**Objective:**

This study aimed to assess the knowledge, attitudes, and practices (KAP) related to dietary nutrition among patients with liver cirrhosis.

**Methods:**

A cross-sectional survey was conducted at July 2025 at The Affiliated Hospital of Guizhou Medical University. Participants were patients clinically diagnosed with liver cirrhosis. Data were collected through a structured questionnaire that included items on demographic information, alongside nutritional knowledge, attitudes toward dietary management, and self-reported dietary behaviors.

**Results:**

A total of 450 valid responses were obtained, yielding a response rate of 97.19%. Among the respondents, 318 (70.7%) were male, 233 (51.8%) had a body mass index (BMI) within the normal range, and 127 (28.2%) had been living with cirrhosis for more than 3 years. The knowledge, attitude, and practice scores were 10.96 ± 3.12 (possible range: 0–26), 21.94 ± 2.94 (possible range: 8–40), and 26.52 ± 4.85 (possible range: 8–40), respectively. The structural equation modeling (SEM) showed that knowledge had direct effects on attitude (*β* = 0.630, *p* < 0.001) and practice (*β* = 1.173, *p* < 0.001). However, neither the direct effect of attitude on practice nor the indirect effect of knowledge on practice was significant.

**Conclusion:**

Individuals diagnosed with liver cirrhosis demonstrated limited knowledge, suboptimal attitudes, and insufficient dietary practices related to nutrition. Given these findings, targeted nutritional education should be integrated into routine clinical care to support behavior change and improve dietary self-management in this population.

## 1 Introduction

Liver cirrhosis represents a significant global health burden, ranking as the 11th most common cause of death worldwide and claiming approximately one million lives annually (1). In China, cirrhosis accounted for 152,261.88 deaths and 4,343,006.24 disability-adjusted life years (DALYs) in 2019, demonstrating the substantial impact of this disease on national public health (2). The disease is frequently accompanied by malnutrition, with prevalence rates of 40–70% in patients with decompensated cirrhosis and 10–40% in those with compensated or asymptomatic cirrhosis (3–5). Malnutrition in cirrhosis is not merely an comorbidity but significantly impacts disease progression and worsens patient prognosis, making nutritional management a critical component of comprehensive care (6).

Nutritional therapy represents one of the few modifiable factors that can positively influence outcomes in cirrhosis patients. Current evidence-based recommendations suggest an energy intake of 30–40 kcal/kg/day and protein intake of 1.0–1.5 g/kg/day for cirrhosis patients, with adjustments based on malnutrition severity (7). However, research indicates that most cirrhosis patients fail to meet these nutritional requirements. A study showed that even patients classified as well-nourished consumed only 76% of recommended caloric intake and 67% of recommended protein intake. Multiple factors impede optimal nutrition, including poor appetite (27%), early satiety (30%), abdominal fullness (25%), dissatisfaction with low-sodium diets (21%), and social myths regarding appropriate diets for liver disease (17%) (8).

Despite the recognized importance of nutrition in cirrhosis management, there remains a significant gap in research exploring patients’ understanding and implementation of dietary guidelines, particularly in the Chinese context. The Knowledge, Attitude, and Practice (KAP) survey approach serves as a valuable diagnostic tool to assess patients’ comprehension, beliefs, and behaviors regarding nutritional management, working on the premise that enhanced knowledge positively influences attitudes, which subsequently shape health behaviors (9, 10). Previous research has shown that many patients unnecessarily restrict protein intake due to misconceptions about its relationship with hepatic encephalopathy, despite evidence not supporting such restrictions (8). Additionally, combined diet and exercise interventions have demonstrated the greatest potential for improving body composition and clinical outcomes in cirrhosis patients, yet adherence to these integrated approaches remains challenging (11) In the Chinese clinical environment, access to structured nutritional counseling for cirrhotic patients remains limited, and misconceptions regarding protein restriction and dietary management are still common (12). To date, no study in China has systematically characterized patient-level nutritional knowledge, attitudes, and daily dietary behaviors using validated tools, nor examined how these elements interact through a structural-equation framework. By mapping the specific knowledge gaps and behavioral barriers in this population, our findings provide evidence that can directly inform the design of targeted, clinically practical nutrition-education strategies. Therefore, this study aims to assess the knowledge, attitudes, and practices of liver cirrhosis patients regarding dietary nutrition in China, with the objective of identifying knowledge gaps, understanding attitudinal barriers, and documenting current nutritional practices to inform the development of tailored educational interventions and improve patient outcomes.

## 2 Methods

### 2.1 Study design and subjects

This cross-sectional study was conducted at July 2025 in The Affiliated Hospital of Guizhou Medical University. A convenience sampling method was employed to recruit individuals diagnosed with liver cirrhosis as study participants. Ethical approval was obtained from the Medical Ethics Committee of The Affiliated Hospital of Guizhou Medical University (2025067K), and written informed consent was secured from all participants prior to data collection.

Inclusion criteria were as follows: (1) patients with medical records of liver cirrhosis; (2) age ≥ 18 years old; (3) voluntary participation.

Exclusion criteria included: (1) patients who were unable to complete the questionnaire due to impaired consciousness; (2) patients who provided incomplete questionnaire.

All enrolled patients had been clinically diagnosed with liver cirrhosis based on the diagnostic criteria outlined in the *Chinese consensus on clinical diagnosis and therapy of liver cirrhosis* (13), with liver biopsy as the “gold standard.”

### 2.2 Questionnaire

The questionnaire was developed based on established clinical guidelines, including *“Chinese consensus on clinical diagnosis and therapy of liver cirrhosis”* and *“Nutrition assessment and diet management in patients with liver cirrhosis”* (14, 15). Following the initial drafting phase, revisions were made based on expert feedback obtained from four senior professionals (specialist in Infectious Diseases Department, Gastroenterology Department, Hepatological Department, Nutrition Department, respectively). To ensure clarity and feasibility, a pilot test was subsequently conducted on a small sample population.

The finalized version of the questionnaire (Supplementary material), administered in Chinese, comprises four main sections: demographic information, knowledge, attitudes, and practices. The knowledge section includes 13 items designed to assess participants’ understanding of nutritional principles relevant to liver cirrhosis. Responses are scored as follows: 2 points for fully correct answers, 1 point for partially correct responses on multiple-choice items, and 0 points for incorrect answers. The total score for this section ranges from 0 to 26. The attitude dimension consists of 8 items evaluated using a five-point Likert scale, with responses ranging from 1 (strongly disagree) to 5 (strongly agree), yielding a total score between 8 and 40. The practice dimension also comprises 8 items. The first two are dichotomous (“Yes” or “No”) and are scored as 5 and 1 point, respectively. Items 3 through 8 utilize a five-point Likert scale with scoring consistent with the attitude section. The cumulative score for the practice domain likewise ranges from 8 to 40.

For all three domains, a score exceeding 70% of the maximum possible value was interpreted as indicating adequate knowledge, a positive attitude, or proactive practice, respectively (16).

During the study period, patients with a recorded diagnosis of liver cirrhosis in the Electronic Medical Record (EMR) were invited to participate. Invitations were extended by distributing quick-response (QR) codes linked to an electronic questionnaire or by handing out paper-based questionnaires face-to-face. The questionnaires were administered by trained clinical nurses. Completion typically required approximately 10–15 min. Participants completed the questionnaire independently; however, brief clarification of item meaning was permitted when requested to reduce misunderstanding while avoiding leading responses.

### 2.3 Questionnaire reliability and validity

During the questionnaire design, the content validity was assessed by a panel of four experts, who reviewed each item for relevance and appropriateness. Nevertheless, no standardized quantitative indices such as the content validity index (CVI) were calculated in this study. In the pilot study, the internal consistency of the questionnaire was evaluated using Cronbach’s *α*. Cronbach’s α was 0.817 for the overall scale, and 0.682, 0.654, and 0.638 for the knowledge, attitude, and practice domains, respectively.

The reliability and validity were also evaluated based on questionnaires collected during formal survey. The Cronbach’s *α* was 0.7272 for the full scale. Item–total correlations were positive and within acceptable ranges. A confirmatory factor analysis (CFA) was conducted (Supplementary Figure S1), and the result yielded RMSEA = 0.060 and SRMR = 0.073, while TLI (0.762) and CFI (0.789) did not reach recommended thresholds, suggesting that the three-factor structure was only partially supported and should be interpreted cautiously. No substantial floor or ceiling effects were identified, as fewer than 15% of participants achieved the minimum or maximum scores in any domain.

### 2.4 Statistical methods

All statistical analyses were performed using SPSS version 22.0 (IBM Corp., Armonk, NY, USA), Stata version 18.0 (StataCorp, College Station, TX, USA), and R version 4.3.2. There were no missing data. Continuous variables were tested for normality. Normally distributed data were presented as means ± standard deviations (SD), and compared by independent samples t-tests for two groups and analysis of variance (ANOVA) for three or more groups. Categorical variables (demographic characteristics and item-level responses) were analyzed descriptively, and presented as *n* (%).

Correlations among the knowledge, attitude, and practice scores were examined using Pearson’s correlation coefficient. Linear regression models were used to examine factors associated with continuous KAP scores. This approach ensured analytical coherence with the SEM framework and avoided information loss from median splits. Structural equation modeling (SEM) was conducted to assess the theoretical pathways among knowledge, attitude, and practice based on the KAP framework. The final sample size (*N* = 450) met common recommendations for SEM, providing more than 10–20 participants per estimated parameter and exceeding the minimum sample size of 200 generally considered adequate. Specifically, the mediating effect of attitude on the relationship between knowledge and practice was evaluated. Bootstrapping with 1,000 resamples was used to obtain the 95% confidence intervals for indirect effects in the SEM. The model fit was assessed using standard indices: the root mean square error of approximation (RMSEA < 0.08), standardized root mean square residual (SRMR < 0.08), Tucker–Lewis index (TLI > 0.80), and comparative fit index (CFI > 0.80). All statistical tests were two-tailed, and *p* < 0.05 was considered statistically significant.

## 3 Results

### 3.1 Demographic characteristics

Initially, a total of 463 samples were collected. The following samples were excluded: two samples lacked informed consent and eleven samples were excluded due to logical errors identified during quality control. The final valid data consisted of 450 samples, with an effective rate of 97.19%. Among them, 318 (70.7%) were male, 144 (32.0%) were aged 65 years and above, 233 (51.8%) had BMI in the normal range, 208 (46.2%) lived in rural areas, 105 (23.3%) were ethnic minorities, 234 (52.0%) had middle school/high/vocational school education, 127 (28.2%) suffered from cirrhosis for more than 3 years, and only 4 (0.8%) were clear about their severity classification of cirrhosis. The knowledge, attitude, and practice scores were 10.96 ± 3.12 (possible range: 0–26), 21.94 ± 2.94 (possible range: 8–40), and 26.52 ± 4.85 (possible range: 8–40), respectively. Differences in knowledge scores were more likely to be found among participants by residence category (*p* = 0.001), education (*p* < 0.001), monthly income per capita (*p* < 0.001), and duration of cirrhosis (*p* = 0.007). Also, differences in attitude and practice scores were more likely to be found among those with different BMI (*p* = 0.041 and *p* = 0.011) and duration of cirrhosis (*p* = 0.035 and *p* = 0.007) (Table 1).

**Table 1:** Demographic characteristics.

*N* = 450	*N* (%)	Knowledge	Effect size	*p*	Attitude	Effect size	*p*	Practice	Effect size	*p*
		mean (SD)	η^2^/Cohen’s d		mean (SD)	η^2^/Cohen’s d		mean (SD)	η^2^/Cohen’s d	
Total score	450 (100.0)	10.96 (3.12)			21.94 (2.94)			26.52 (4.85)		
Age, years			4.97 × 10^−4^	0.855		4.18 × 10^−4^	0.851		9.81 × 10^−4^	0.644
18–54	166 (36.9)	11.02 (3.31)			22.04 (3.03)			26.30 (5.06)		
55–64	140 (31.1)	11.01 (2.91)			21.86 (2.82)			26.66 (4.61)		
65 and more	144 (32.0)	10.85 (3.10)			21.90 (2.97)			26.65 (4.84)		
Gender			0.05	0.459		0.09	0.322		0.10	0.321
Male	318 (70.7)	11.01 (3.02)			21.86 (2.99)			26.66 (4.85)		
Female	132 (29.3)	10.84 (3.35)			22.13 (2.81)			26.19 (4.85)		
BMI, kg/m^2^			0.01	0.170		0.01	**0.041**		0.01	**0.011**
<18.50	77 (17.1)	10.65 (3.00)			22.79 (2.86)			28.12 (4.80)		
18.50–23.99	233 (51.8)	10.71 (2.90)			21.88 (2.90)			26.24 (4.91)		
24.00–27.99	114 (25.3)	11.54 (3.53)			21.49 (2.94)			26.01 (4.68)		
> = 28.00	26 (5.8)	11.58 (3.14)			21.85 (3.11)			26.62 (4.39)		
Residence			0.03	**0.001**		0.01	0.073		9.84 × 10^−3^	0.052
Rural	208 (46.2)	10.39 (2.85)			21.65 (2.92)			26.21 (4.98)		
Urban	185 (41.1)	11.35 (3.38)			22.02 (2.96)			26.43 (4.81)		
Suburban	57 (12.7)	11.75 (2.82)			22.70 (2.83)			27.98 (4.28)		
Ethnicity			0.18	0.133		0.18	0.209		0.11	0.317
Han	345 (76.7)	11.09 (3.18)			22.06 (3.00)			26.65 (4.88)		
Ethnic minority	105 (23.3)	10.52 (2.87)			21.52 (2.72)			26.11 (4.73)		
Education			0.05	**<0.001**		4.13 × 10^−3^	0.216		3.32 × 10^−3^	0.350
Primary school or below	130 (28.9)	10.14 (2.81)			21.77 (3.19)			26.26 (5.14)		
Middle school/High school/Vocational school	234 (52.0)	10.93 (2.87)			21.88 (2.86)			26.45 (4.57)		
Associate degree/Bachelor’s degree or above	86 (19.1)	12.29 (3.73)			22.36 (2.73)			27.13 (5.14)		
Monthly income, CNY			0.08	**<0.001**		9.61 × 10^−4^	0.263		9.78 × 10^−3^	0.064
<5,000	191 (42.4)	10.16 (2.59)			21.92 (3.00)			25.95 (5.07)		
5,000–10,000	205 (45.6)	11.18 (3.09)			22.10 (2.81)			26.88 (4.62)		
>10,000	54 (13.0)	12.96 (3.87)			21.37 (3.18)			27.19 (4.79)		
Duration of cirrhosis			0.24	**0.007**		0.20	**0.035**		0.27	**0.007**
3 months or less	144 (32.0)	10.19 (2.66)			21.38 (2.68)			25.42 (4.77)		
4 months – 1 year	67 (14.9)	11.09 (3.22)			22.61 (3.48)			26.54 (4.91)		
1 year – 3 years	112 (24.9)	11.06 (3.06)			22.18 (3.06)			26.99 (4.61)		
More than 3 years	127 (28.2)	11.67 (3.43)			22.00 (2.72)			27.36 (4.94)		

Percentages are calculated using the total sample size (*N* = 450). Knowledge, attitude, and practice scores range from 0–26, 8–40, and 8–40, respectively. A score >70% of the domain maximum was defined as “adequate.” The effect sizes were expressed as η^2^ (comparison among three groups or more) or Cohen’s d (comparison between two groups). Conventional cutoff: η^2^: 0.01 (small), 0.06 (medium), 0.14 (large); Cohen’s d: 0.2 (small), 0.5 (medium), 0.8 (large). *P* < 0.05 was marked as bold.

### 3.2 Distribution of responses to knowledge, attitude, and practice

Responses to the knowledge dimension showed that only 23.11% were aware of the “Dietary Guidelines for Chinese Residents,” the “Dietary Pyramid for Chinese Residents,” or other dietary nutrition recommendations (K1). Regarding nutritional evaluation methods, only 30.44% were aware of measuring height, weight, and grip strength (K8.a). Notably, 62% were not sure which nutrients needed to be restricted when ascites or edema occurs in cirrhosis (K12.d) (Table 2).

**Table 2:** Distribution of knowledge dimension responses.

Knowledge	Option a Yes/Selected	Option b No/Selected	Option c Selected	Option d Selected
1. Have you ever learned about the “Dietary Guidelines for Chinese Residents,” the “Dietary Pyramid for Chinese Residents,” or other dietary nutrition recommendations?	104 (23.11%)	346 (76.89%)		
2. The six essential nutrients for the human body include carbohydrates, fats, proteins, minerals, vitamins, and water. To meet the body’s needs, what foods should we eat? (a. Grains and tubers)	429 (95.33%)	21 (4.67%)		
2 (b. Fish, poultry, eggs, lean meat, dairy products, beans, and bean products)	427 (94.89%)	23 (5.11%)		
2 (c. Vegetables and fruits)	401 (89.11%)	49 (10.89%)		
2 (d. Water)	165 (36.67%)	285 (63.33%)		
2 (e. Oil)	146 (32.44%)	304 (67.56%)		
2 (f. Sugar)	39 (8.67%)	411 (91.33%)		
2 (h. Not sure)	9 (2%)	441 (98%)		
3. For healthy individuals, ensuring a daily intake of at least ___ ml of water is beneficial for health.	84 (18.67%)	143 (31.78%)	27 (6%)	196 (43.56%)
4. Malnutrition is one of the complications of cirrhosis. Which of the following factors make cirrhosis patients more prone to malnutrition? (a. Lack of appetite, unable to eat)	424 (94.22%)	26 (5.78%)		
4 (b. Poor digestion/absorption function;)	283 (62.89%)	167 (37.11%)		
4 (c. Increased body expenditure)	159 (35.33%)	291 (64.67%)		
4 (d. Side effects of medications)	84 (18.67%)	366 (81.33%)		
4 (e. Decline in liver metabolism function)	38 (8.44%)	412 (91.56%)		
4 (f. Other)	30 (6.67%)	420 (93.33%)		
5. Malnutrition increases the risk of other complications in cirrhosis patients, including: (a. Edema)	134 (29.78%)	316 (70.22%)		
5 (b. Ascites)	159 (35.33%)	291 (64.67%)		
5 (c. Infection)	314 (69.78%)	136 (30.22%)		
5 (d. Other)	96 (21.33%)	354 (78.67%)		
6. Signs of malnutrition in cirrhosis patients include (a. Weakness)	404 (89.78%)	46 (10.22%)		
6 (b. Muscle loss)	85 (18.89%)	365 (81.11%)		
6 (c. Weight loss)	362 (80.44%)	88 (19.56%)		
6 (d. Other)	14 (3.11%)	436 (96.89%)		
7. Cirrhosis can cause protein metabolism disorders. Increasing intake of high-quality proteins helps protect liver function. Which of the following foods are rich in high-quality protein? (a. Fish, poultry, lean meat, eggs, dairy products)	417 (92.67%)	33 (7.33%)		
7 (b. Soybeans and their products)	179 (39.78%)	271 (60.22%)		
7 (c. Grains, tubers, and vegetables)	39 (8.67%)	411 (91.33%)		
7 (d. Other)	15 (3.33%)	435 (96.67%)		
8. Cirrhosis patients need nutritional screening and assessment. Are you aware of (or have you undergone) the following commonly used nutritional evaluation methods for cirrhosis patients? (a. Measuring height, weight, and grip strength)	137 (30.44%)	313 (69.56%)		
8 (b. Nutritionist conducting body composition analysis)	43 (9.56%)	407 (90.44%)		
8 (c. Blood tests to check albumin levels)	344 (76.44%)	106 (23.56%)		
8 (d. Nutritionist asking dietary-related questions for evaluation)	79 (17.56%)	371 (82.44%)		
8 (e. None of above)	92 (20.44%)	358 (79.56%)		
9. Protein intake is very important for cirrhosis patients, but in the presence of the following complications, protein intake should be reduced or restricted:	7 (1.56%)	16 (3.56%)	11 (2.44%)	416 (92.44%)
10. In addition to protein, cirrhosis patients need to ensure the intake of the following nutrients: (a. Grains and tubers)	268 (59.56%)	182 (40.44%)		
10 (b. Fats)	16 (3.56%)	434 (96.44%)		
10 (c. Vegetables and fruits)	159 (35.33%)	291 (64.67%)		
10 (d. Not sure)	179 (39.78%)	271 (60.22%)		
11. The following eating habits are suitable for cirrhosis patients: (a. Eating smaller meals more frequently)	420 (93.33%)	30 (6.67%)		
11 (b. Having a snack before bed)	128 (28.44%)	322 (71.56%)		
11 (c. Binge eating)	40 (8.89%)	410 (91.11%)		
12. When ascites or edema occurs in cirrhosis, the intake of the following nutrients should be restricted: (a. Sodium (e.g., salt))	117 (26%)	333 (74%)		
12 (b. Water)	121 (26.89%)	329 (73.11%)		
12 (c. Sugar)	6 (1.33%)	444 (98.67%)		
12 (d. Not sure)	279 (62%)	171 (38%)		
13. The following are part of the nutritional support treatment for cirrhosis patients: (a. Antibiotic treatment)	2 (0.44%)	448 (99.56%)		
13 (b. Parenteral nutrition)	250 (55.56%)	200 (44.44%)		
13 (c. Enteral nutrition)	281 (62.44%)	169 (37.56%)		
13 (d. Not sure)	136 (30.22%)	314 (69.78%)		

Percentages are calculated using the total sample size (*N* = 450).

Responses to the attitude dimension showed that when it comes to whether having adequate knowledge about the nutritional content of different foods (A1) and dietary needs in cirrhosis (A2), 23.11 and 19.56% were very non-confident and 57.3 and 44.22% were non-confident. On the other hand, when it comes to following a strictly prescribed diet every day (A5) and ensuring regular and timely meals (A6), 6.67 and 8.22% find it very difficult, while 28.89 and 26.67% find it difficult to do so (Table 3).

**Table 3:** Distribution of attitude dimension responses.

Attitude	Strongly agree	Agree	Neutral	Disagree	Strongly disagree
1. I am confident that I have a thorough understanding of the nutritional content of different foods.	6 (1.33%)	36 (8%)	46 (10.22%)	258 (57.33%)	104 (23.11%)
2. I am confident that I have a thorough understanding of the dietary needs for cirrhosis.	5 (1.11%)	90 (20%)	68 (15.11%)	199 (44.22%)	88 (19.56%)
3. I believe that ensuring an appropriate diet is very important for my disease recovery.	32 (7.11%)	351 (78%)	48 (10.67%)	15 (3.33%)	4 (0.89%)
4. I believe that dietary nutrition and medication treatment are equally important for my disease recovery.	36 (8%)	319 (70.89%)	83 (18.44%)	12 (2.67%)	0 (0%)
5. I believe that it is difficult to follow a strictly prescribed diet every day.	30 (6.67%)	130 (28.89%)	154 (34.22%)	120 (26.67%)	16 (3.56%)
6. I believe that it is difficult to ensure I eat on time every day.	37 (8.22%)	120 (26.67%)	151 (33.56%)	121 (26.89%)	21 (4.67%)
7. I believe that my disease sometimes affects my appetite.	92 (20.44%)	296 (65.78%)	51 (11.33%)	9 (2%)	2 (0.44%)
8. I believe that changing unhealthy habits like smoking and drinking alcohol can help with my disease recovery.	32 (7.11%)	242 (53.78%)	147 (32.67%)	27 (6%)	2 (0.44%)

Percentages are calculated using the total sample size (*N* = 450).

Responses to the practice dimension showed that 31.56% never participate in education related to dietary and nutritional needs (P1), 43.56% never undergo a nutritional assessment (P2), and 50.22% never supplement their diet through supplements or other means (P8) (Table 4).

**Table 4:** Distribution of practice dimension responses.

Practice	Yes/Always	No/Often	Neutral	Rarely	Never
1. Have you participated in or are you currently participating in any education related to dietary and nutritional needs?	308 (68.44%)	142 (31.56%)			
2. Have you undergone a nutritional assessment?	254 (56.44%)	196 (43.56%)			
	Always	Often	Neutral	Rarely	Never
3. Do you follow the diet prescribed by your doctor at every meal?	37 (8.22%)	277 (61.56%)	106 (23.56%)	22 (4.89%)	8 (1.78%)
4. Are you able to follow the practice of eating smaller, more frequent meals in your daily diet?	20 (4.44%)	137 (30.44%)	195 (43.33%)	83 (18.44%)	15 (3.33%)
5. How often do you include carbohydrates such as rice or noodles in your daily diet?	121 (26.89%)	228 (50.67%)	92 (20.44%)	8 (1.78%)	1 (0.22%)
6. How often do you include protein-rich foods such as lean meat, soy products, dairy products, or fish in your daily diet?	47 (10.44%)	166 (36.89%)	201 (44.67%)	35 (7.78%)	1 (0.22%)
7. How often do you include vitamin- and fiber-rich foods such as vegetables and fruits in your daily diet?	32 (7.11%)	169 (37.56%)	205 (45.56%)	44 (9.78%)	0 (0%)
8. How often do you supplement your diet with amino acids, trace elements, vitamins, or other nutritional support through supplements or other means?	5 (1.11%)	25 (5.56%)	50 (11.11%)	144 (32%)	226 (50.22%)

Percentages are calculated using the total sample size (*N* = 450).

### 3.3 Univariate and multivariate analysis of knowledge, attitude, and practice

Multivariate linear regression showed that knowledge scores were independently associated with several demographic variables. Compared with rural residents, those living in suburban areas had higher knowledge scores (*β* = 1.132, 95% CI: 0.257–2.006, *p* = 0.012). Higher education was also associated with higher knowledge, particularly among participants with an associate degree or above (*β* = 1.321, 95% CI: 0.403–2.238, *p* = 0.005). Monthly income demonstrated a strong positive association, with those earning more than 10,000 CNY per month showing higher knowledge scores (*β* = 1.966, 95% CI: 0.942–2.990, *p* < 0.001). In addition, longer disease duration was positively associated with knowledge, especially in participants living with cirrhosis for more than three years (*β* = 1.016, 95% CI: 0.293–1.738, *p* = 0.006) (Supplementary Table S1).

For attitude scores, multivariate analysis indicated that knowledge remained an independent positive predictor (*β* = 0.233, 95% CI: 0.147–0.319, *p* < 0.001). BMI showed a negative association, with participants in the 18.50–23.99 kg/m^2^ group (*β* = −0.955, 95% CI: −1.677, −0.233, *p* = 0.010) and the 24.00–27.99 kg/m^2^ group (*β* = −1.552, 95% CI: −2.366, −0.739, *p* < 0.001) reporting lower attitude scores compared with underweight participants. Disease duration of 4 months–1 year was also associated with more positive attitudes (*β* = 1.062, 95% CI: 0.246–1.879, *p* = 0.011) (Supplementary Table S2).

For practice scores, both knowledge (*β* = 0.374, 95% CI: 0.224–0.523, *p* < 0.001) and attitude (*β* = 0.144, 95% CI: −0.010–0.298, *p* = 0.067) showed positive associations, though the latter did not reach statistical significance. BMI again showed a negative association: participants with BMI 18.50–23.99 (*β* = −1.901, 95% CI: −3.088, −0.714, *p* = 0.002) and 24.00–27.99 (β = −2.463, 95% CI: −3.810, −1.116, *p* < 0.001) reported lower practice scores. Longer disease duration was associated with better practice, particularly among those with more than 3 years of illness (*β* = 1.333, 95% CI: 0.204–2.462, *p* = 0.021) (Supplementary Table S3).

### 3.4 Correlations and SEM analysis

**Figure 1:**
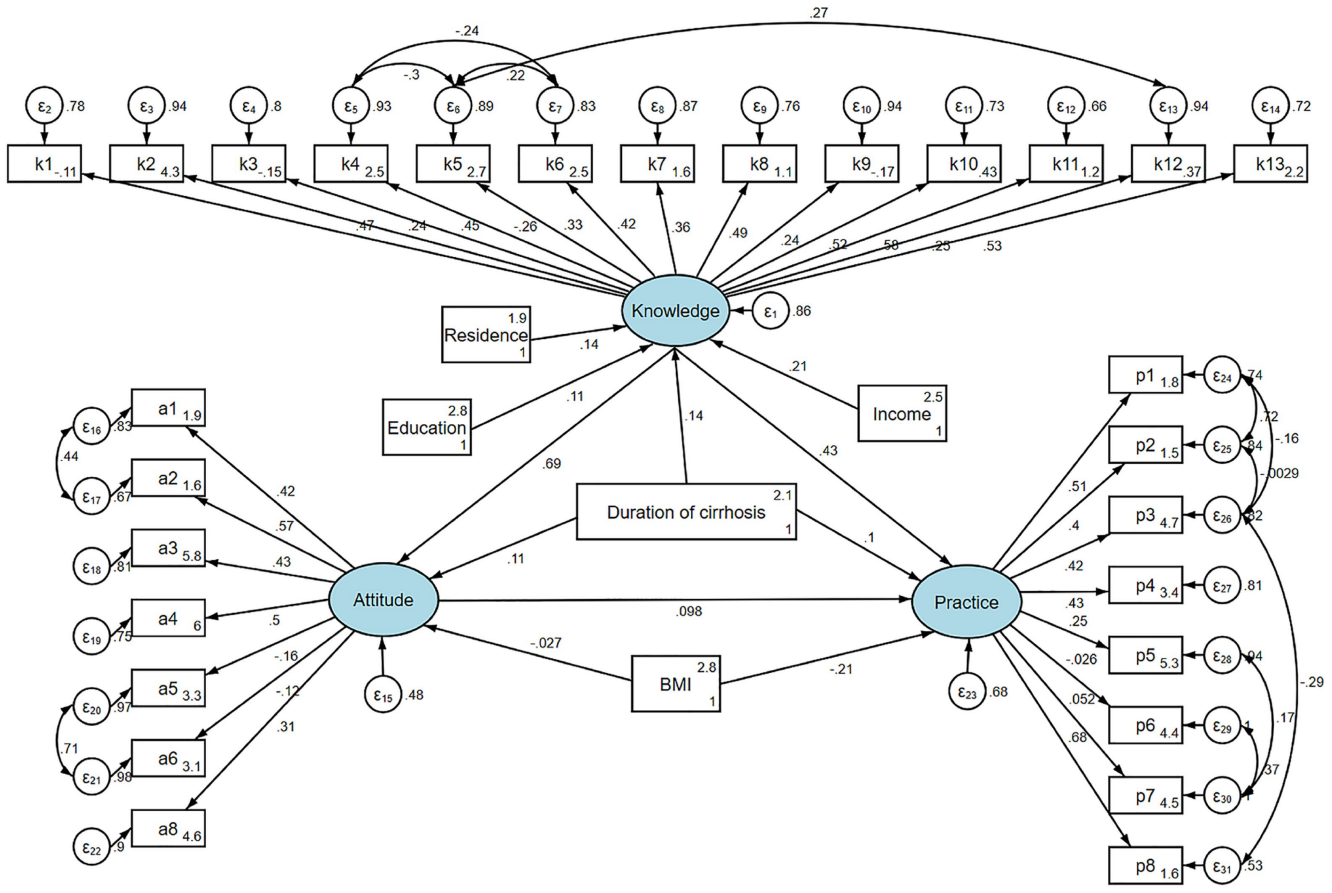
SEM model.

Correlation analysis showed significant positive relationships between knowledge and attitude (*r* = 0.276, *p* < 0.001), knowledge and practice (*r* = 0.278, *p* < 0.001), and attitude and practice (*r* = 0.181, *p* < 0.001) (Supplementary Table S4). Structural equation modeling further examined these pathways (Figure 1). After adjusted for demographic characteristics that showed statistic difference in multivariate linear regression, knowledge demonstrated a strong direct effect on attitude (*β* = 0.630, 95% CI: 0.388–0.872) and a substantial total effect on practice (*β* = 1.173, 95% CI: 0.707–1.639), while the indirect effect through attitude was not statistically significant. Attitude did not show a significant direct effect on practice (*β* = 0.253, 95% CI: −0.436–0.942) (Table 5).

**Table 5:** Results of structural equation model.

Model paths	Total effect		Direct effect		Indirect effect	
	β (95%CI)	*p*	β (95%CI)	*p*	β (95%CI)	*p*
Knowledge						
Residence	0.059 (0.013, 0.105)	0.011	0.059 (0.013, 0.105)	0.011		
Education	0.065 (0.000, 0.131)	0.052	0.065 (0.000, 0.131)	0.052		
Income	0.122 (0.051, 0.193)	0.001	0.122 (0.051, 0.193)	0.001		
Duration of cirrhosis	0.048 (0.012, 0.084)	0.010	0.048 (0.012, 0.084)	0.010		
Attitude						
Knowledge	0.630 (0.388, 0.872)	<0.001	0.630 (0.388, 0.872)	<0.001		
Residence	0.037 (0.007, 0.068)	0.017			0.037 (0.007, 0.068)	0.017
Education	0.041 (−0.002, 0.084)	0.059			0.041 (−0.002, 0.084)	0.059
Income	0.077 (0.029, 0.124)	0.002			0.077 (0.029, 0.124)	0.002
BMI	−0.013 (−0.065, 0.040)	0.641	−0.013 (−0.065, 0.040)	0.641		
Duration of cirrhosis	0.065 (0.024, 0.107)	0.002	0.035 (−0.002, 0.072)	0.063	0.030 (0.006, 0.054)	0.014
Practice						
Knowledge	1.173 (0.707, 1.639)	<0.001	1.014 (0.376, 1.652)	0.002	0.159 (−0.270, 0.588)	0.466
Attitude	0.253 (−0.436, 0.942)	0.472	0.253 (−0.436, 0.942)	0.472		
Residence	0.069 (0.011, 0.127)	0.019			0.069 (0.011, 0.127)	0.019
Education	0.076 (−0.002, 0.155)	0.057			0.076 (−0.002, 0.155)	0.057
Income	0.143 (0.053, 0.233)	0.002			0.143 (0.053, 0.233)	0.002
BMI	−0.255 (−0.392, −0.119)	<0.001	−0.252 (−0.388, -0.116)	<0.001	−0.003 (−0.019, 0.013)	0.697
Duration of cirrhosis	0.148 (0.055, 0.240)	0.002	0.082 (−0.004, 0.169)	0.063	0.065 (0.015, 0.115)	0.011

Model fit indices indicated acceptable RMSEA (0.054) and SRMR (0.069), whereas TLI (0.749) and CFI (0.774) did not reach commonly recommended thresholds. As the TLI and CFI values did not meet commonly accepted cutoffs, these findings should be interpreted with caution, as the suboptimal model fit may reflect measurement limitations or heterogeneity within the study population.

## 4 Discussion

Patients with liver cirrhosis demonstrated suboptimal levels of nutritional knowledge, which were significantly associated with less favorable attitudes and substandard dietary practices. These findings highlight the need for integrating individualized nutritional education into routine clinical management to enhance patients’ engagement in effective dietary self-care.

These findings are consistent with previous observations that limited knowledge and lack of confidence can hinder the formation of positive health attitudes and prevent the adoption of effective self-management behaviors in patient populations (17). This gap between perceived importance and practical application reflects a broader challenge in patient education and health behavior change, where intention is not always supported by the necessary informational or environmental resources. Studies conducted in tertiary care settings have also suggested that cirrhosis patients are less likely to receive formal dietary counseling unless symptoms become severe, a trend that may partly explain the low exposure to foundational concepts such as dietary guidelines or structured assessment tools observed in our cohort (18, 19). For instance, few patients were aware of national dietary guidelines, and many lacked understanding of basic nutritional assessment methods. Additionally, there was widespread uncertainty about which nutrients should be limited when complications such as ascites or edema occur, reflecting significant deficiencies in disease-specific dietary knowledge.

Although the three domains were positively correlated, the structural equation model showed that the pathway structure differed from the classical KAP sequence. Knowledge exerted a strong direct effect on attitude and remained the main driver of practice; however, the indirect pathway through attitude was not statistically significant. Attitude itself did not show a significant direct effect on practice, suggesting that acknowledging the importance of nutrition may not translate into daily dietary behaviors in this population. In the revised analysis, all regression models were standardized by using linear regression so that all variables remained continuous. Consistent with the updated SEM, the linear regression results also showed that the attitude practice association was not statistically significant, resolving the discrepancy observed in the previous version. This pattern is consistent with settings in which external factors—such as financial constraints, symptom burden, or limited access to dietary counseling—may limit the ability of patients to act on their intentions.

Sociodemographic variables further illustrated this constraint. Higher income, longer disease duration, and higher education level were associated with more favorable KAP scores, whereas higher BMI was negatively associated with practice. These findings indicate that behavior is shaped not only by knowledge but also by patients’ physical condition and socioeconomic context. In this context, inadequate knowledge and ability-related limitations represent distinct barriers. Some patients may understand dietary recommendations but remain unable to follow them due to symptom burden, BMI-related functional limitations, or socioeconomic constraints. Together, these results highlight that improving knowledge alone may not be sufficient unless accompanied by practical support and accessible dietary guidance. Moreover, some barriers to dietary adherence may arise not only from limited knowledge but also from physical or socioeconomic constraints, such as symptom burden, reduced functional capacity, or restricted access to suitable food options, which may prevent patients from implementing recommended practices even when they understand them. As the TLI and CFI values did not meet commonly accepted thresholds, these findings should be interpreted with caution, as the suboptimal model fit may reflect measurement limitations or heterogeneity within the study population (20). Despite incorporating covariates, the SEM yielded TLI and CFI values below recommended thresholds, indicating that the model structure should be interpreted cautiously even after revision. Several factors in this study may have contributed to the suboptimal fit. The internal consistency of the three subscales was modest, and the questionnaire did not undergo full psychometric validation beyond Cronbach’s *α* and CFA. In addition, the sample showed notable heterogeneity across BMI, education, and income, and the single-center convenience sampling may have limited the stability of the model structure.

Our multivariate analysis also revealed several sociodemographic factors that shape KAP performance. Suburban residence, higher education level, higher income, and longer disease duration were all positively associated with better knowledge scores, indicating that individuals with stronger socioeconomic resources or longer clinical experience may have more opportunities to receive nutritional information. For attitudes, knowledge emerged as the primary predictor, while BMI showed a negative association and disease duration of 4 months to 1 year was linked to more positive attitudes. In the practice domain, knowledge showed a clear positive association, whereas the effect of attitude did not reach statistical significance. BMI again demonstrated a negative association, with patients in the normal or slightly elevated range reporting better practices than their underweight counterparts. This pattern aligns with clinical observations: patients who are underweight often experience fatigue, gastrointestinal symptoms, or reduced functional capacity, which can hinder their ability to implement dietary recommendations even when they understand them. These findings highlight that effective dietary management depends not only on education but also on patients’ physical status and living conditions, which may constrain the translation of knowledge into action.

A closer examination of the item-level responses across the three dimensions reveals additional insights. Within the knowledge section, patients demonstrated familiarity with general nutrition concepts but lacked clarity on specific, clinically relevant details—such as water intake recommendations or the nuanced roles of different nutrients in cirrhosis management. This suggests that while broad public health messaging may have reached the patient population, condition-specific knowledge remains insufficient. Such patterns have been reported in other chronic disease contexts where patients can correctly identify basic food groups but struggle to apply this knowledge in the setting of disease-specific restrictions (21, 22). Patient attitudes reflected a disconnect between recognizing the importance of nutrition and feeling equipped to act on that knowledge. Many expressed limited confidence in their understanding of appropriate dietary choices for cirrhosis and reported difficulties in consistently adhering to dietary routines such as meal timing or prescribed restrictions (23). In terms of dietary practices, many patients had not participated in formal nutrition education or undergone professional assessment, and few reported regular use of supplements or consistent adherence to recommended eating patterns. This suggests that proactive dietary management remains underutilized in routine care (24, 25).

The practical implications of these findings point to several system-level and clinical priorities. First, nutritional education should be integrated into standard cirrhosis care pathways, rather than being offered only when malnutrition becomes apparent. This requires restructuring outpatient care to include routine screening and brief dietary interventions at early disease stages. Hospitals should consider embedding trained dietitians into hepatology teams or utilizing telehealth consultations to extend reach in resource-limited areas. Second, educational efforts must be tailored to the literacy level and emotional readiness of the patient. Simply providing information is insufficient; instead, patients may benefit more from scenario-based counseling, culturally adapted dietary models, and printed materials that reflect real-life food choices. Community health workers and primary care providers should be mobilized to deliver this education, especially in rural regions where specialist access is limited. Finally, longer-term improvements will depend on addressing resource allocation, training standards, and monitoring systems. Policymakers should consider incentivizing the incorporation of nutrition metrics into chronic disease care evaluations, while institutions can pilot scalable interventions such as group education sessions or app-based dietary self-management tools (26, 27).

From a clinical operational perspective, a brief and feasible workflow may help integrate nutrition support into routine visits for cirrhosis patients. First, clinicians can conduct a rapid three-item screening focusing on sodium restriction, protein adequacy, and late-evening snacks to identify immediate risks or misconceptions. Second, providing a simple one-page handout summarizing recommended meal patterns, example high-quality protein sources, and key sodium-containing foods can reinforce essential concepts. Third, arranging a short follow-up consultation—such as a 10-min telephone call with a dietitian—may offer patients tailored guidance and prompt clarification of questions that arise during implementation. This pragmatic approach does not imply causality but offers clinicians actionable steps that can be adopted immediately in routine care.

This study has several limitations that should be acknowledged. First, its cross-sectional design restricts the ability to draw causal inferences between knowledge, attitudes, and practices, as temporal sequencing cannot be established. Second, all data were collected through self-reported questionnaires, which may be subject to recall bias or social desirability bias, potentially affecting the accuracy of responses regarding both knowledge and behavior. Third, the sample was drawn from a single institution, and although it included a relatively diverse patient group, the findings may not be generalizable to populations in different regions or healthcare settings with varying access to nutritional services. Although internal consistency and CFA were performed, the questionnaire did not undergo full psychometric validation (e.g., CVI, test–retest reliability) and the Cronbach’s *α* for specific dimension was relative low, which may limit the robustness of score interpretations. Additionally, important clinical descriptors such as Child–Pugh class, MELD score, disease etiology, ascites, hepatic encephalopathy, and use of diuretics or lactulose were not collected. The absence of these variables limits our ability to assess disease severity or adjust for clinical confounding in knowledge, attitude, and practice outcomes. Moreover, only 0.8% of participants reported knowing their severity classification, indicating that self-reported clinical information was extremely limited. This further restricts our ability to interpret dietary behaviors in relation to disease severity. Because the sample was drawn from a single institution using convenience sampling, selection bias cannot be excluded. In particular, if patients of lower socioeconomic status were more likely to seek care at this facility, the sample may not fully reflect the broader cirrhosis population, thereby limiting the external validity of the findings. Future studies should adopt multi-center sampling strategies, include both rural and urban populations, and incorporate exposure to formal dietitian-led education as a key variable to improve generalisability and strengthen inference. Despite these limitations, the study provides meaningful insights into the nutritional perceptions and behaviors of patients with liver cirrhosis, offering a foundation for future research and intervention design aimed at improving dietary management in this population.

In conclusion, individuals diagnosed with liver cirrhosis exhibited suboptimal levels of knowledge, generally passive attitudes, and insufficient engagement in dietary nutrition practices, with significant gaps identified between knowledge and behavior. These findings underscore the need for targeted, knowledge-based nutritional education interventions in clinical settings to enhance both motivation and adherence to dietary recommendations among cirrhotic patients. In addition, because the study was conducted in a single hospital, the findings mainly reflect the characteristics of this local patient population and may not be generalizable to cirrhosis patients across China. In addition, although basic reliability testing was conducted, the questionnaire did not undergo a full standardized validation procedure, and the results should therefore be interpreted with caution.

## Data Availability

The original contributions presented in the study are included in the article/Supplementary material, further inquiries can be directed to the corresponding author.
