# Supplementary material for: Knowledge, attitudes, and practices of liver cirrhosis patients regarding dietary nutrition
Source: Front Nutr. 2026 Jan 6;12:1707256. doi: 10.3389/fnut.2025.1707256 (PMC12815832; doi:10.3389/fnut.2025.1707256)
Supplement: Supplementary file 1 [file Table_1.docx]

**Table S1. Univariate and multivariate linear regression analysis for knowledge**

| **Knowledge** | Univariate analysis |  | Multivariate analysis |  |
| --- | --- | --- | --- | --- |
|  | β (95%CI) | P | β (95%CI) | P |
| **Age** |  |  |  |  |
| 18-54 | REF |  |  |  |
| 55-64 | -0.011 (-0.715,0.693) | 0.976 |  |  |
| 65 and more | -0.171 (-0.870,0.528) | 0.631 |  |  |
| **Gender** |  |  |  |  |
| Male | REF |  |  |  |
| Female | -0.169 (-0.803,0.466) | 0.602 |  |  |
| **BMI** |  |  |  |  |
| <18.50 | REF |  | REF |  |
| 18.50-23.99 | 0.063 (-0.738,0.864) | 0.877 | 0.019 (-0.743,0.781) | 0.960 |
| 24.00-27.99 | 0.886 (-0.013,1.785) | 0.054 | 0.584 (-0.271,1.439) | 0.182 |
| >=28.00 | 0.928 (-0.455,2.310) | 0.188 | 0.632 (-0.687,1.951) | 0.348 |
| **Residence** |  |  |  |  |
| Rural | REF |  | REF |  |
| Urban | 0.957 (0.346,1.568) | 0.002 | 0.223 (-0.433,0.880) | 0.505 |
| Suburban | 1.360 (0.457,2.264) | 0.003 | 1.132 (0.257,2.006) | 0.012 |
| **Ethnicity** |  |  |  |  |
| Han | REF |  |  |  |
| Ethnic minority | -0.569 (-1.250,0.112) | 0.101 |  |  |
| **Education** |  |  |  |  |
| Primary school or below | REF |  | REF |  |
| Middle school/High school/Vocational school | 0.789 (0.136,1.442) | 0.018 | 0.574 (-0.091,1.238) | 0.092 |
| Associate degree/Bachelor’s degree or above | 2.152 (1.323,2.982) | <0.001 | 1.321 (0.403,2.238) | 0.005 |
| **Monthly income per capita** |  |  |  |  |
| <5000 | REF |  | REF |  |
| 5000-10000 | 1.023 (0.432,1.615) | 0.001 | 0.423 (-0.261,1.107) | 0.226 |
| >10000 | 2.806 (1.899,3.713) | <0.001 | 1.966 (0.942,2.990) | <0.001 |
| **Duration of cirrhosis** |  |  |  |  |
| 3 months or less | REF |  | REF |  |
| 4 months – 1 year | 0.895 (0.002,1.788) | 0.049 | 0.711 (-0.154,1.577) | 0.108 |
| 1 year – 3 years | 0.868 (0.107,1.629) | 0.025 | 0.648 (-0.088,1.383) | 0.085 |
| More than 3 years | 1.475 (0.740,2.210) | <0.001 | 1.016 (0.293,1.738) | 0.006 |

All analyses used the full sample (N = 450).

**Table S2. Univariate and multivariate linear regression analysis for attitude**

| **Attitude** | Univariate analysis |  | Multivariate analysis |  |
| --- | --- | --- | --- | --- |
|  | β (95%CI) | P | β (95%CI) | P |
| **Knowledge** | 0.354 (0.021,0.050) | <0.001 | 0.233 (0.147,0.319) | <0.001 |
| **Age** |  |  |  |  |
| 18-54 | REF |  |  |  |
| 55-64 | -0.172 (-0.836,0.492) | 0.611 |  |  |
| 65 and more | -0.140 (-0.799,0.519) | 0.676 |  |  |
| **Gender** |  |  |  |  |
| Male | REF |  |  |  |
| Female | 0.270 (-0.328,0.869) | 0.375 |  |  |
| **BMI** |  |  |  |  |
| <18.50 | REF |  | REF |  |
| 18.50-23.99 | -0.908 (-1.662,-0.154) | 0.018 | -0.955 (-1.677,-0.233) | **0.010** |
| 24.00-27.99 | -1.301 (-2.147,-0.455) | 0.003 | -1.552 (-2.366,-0.739) | **<0.001** |
| >=28.00 | -0.946 (-2.247,0.355) | 0.154 | -1.351 (-2.602,-0.101) | **0.035** |
| **Residence** |  |  |  |  |
| Rural | REF |  | REF |  |
| Urban | 0.368 (-0.213,0.949) | 0.214 | 0.120 (-0.456,0.697) | 0.682 |
| Suburban | 1.048 (0.188,1.908) | 0.017 | 0.780 (-0.059,1.619) | 0.069 |
| **Ethnicity** |  |  |  |  |
| Han | REF |  | REF |  |
| Ethnic minority | -0.540 (-1.183,0.103) | 0.099 | -0.377 (-1.011,0.256) | 0.244 |
| **Education** |  |  |  |  |
| Primary school or below | REF |  |  |  |
| Middle school/High school/Vocational school | 0.107 (-0.525,0.738) | 0.740 |  |  |
| Associate degree/Bachelor’s degree or above | 0.591 (-0.211,1.394) | 0.148 |  |  |
| **Monthly income per capita** |  |  |  |  |
| <5000 | REF |  |  |  |
| 5000-10000 | 0.181 (-0.400,0.761) | 0.540 |  |  |
| >10000 | -0.551 (-1.441,0.339) | 0.224 |  |  |
| **Duration of cirrhosis** |  |  |  |  |
| 3 months or less | REF |  | REF |  |
| 4 months – 1 year | 1.230 (0.382,2.078) | 0.005 | 1.062 (0.246,1.879) | **0.011** |
| 1 year – 3 years | 0.797 (0.074,1.519) | 0.031 | 0.680 (-0.016,1.376) | 0.056 |
| More than 3 years | 0.618 (-0.080,1.316) | 0.083 | 0.309 (-0.371,0.989) | 0.373 |

All analyses used the full sample (N = 450).

**Table S3. Univariate and multivariate linear regression analysis for practice**

| **Practice** | Univariate analysis |  | Multivariate analysis |  |
| --- | --- | --- | --- | --- |
|  | β (95%CI) | P | β (95%CI) | P |
| **Knowledge** | 0.312 (0.166,0.458) | <0.001 | 0.374 (0.224,0.523) | <0.001 |
| **Attitude** | 0.306 (0.152,0.462) | <0.001 | 0.144 (-0.010,0.298) | 0.067 |
| **Age** |  |  |  |  |
| 18-54 | REF |  |  |  |
| 55-64 | 0.369 (-0.726,1.464) | 0.508 |  |  |
| 65 and more | 0.358 (-0.729,1.444) | 0.518 |  |  |
| **Gender** |  |  |  |  |
| Male | REF |  |  |  |
| Female | -0.474 (-1.461,0.512) | 0.345 |  |  |
| **BMI** |  |  |  |  |
| <18.50 | REF |  | REF |  |
| 18.50-23.99 | -1.877 (-3.119,-0.635) | 0.003 | -1.901 (-3.088,-0.714) | **0.002** |
| 24.00-27.99 | -2.108 (-3.502,-0.714) | 0.003 | -2.463 (-3.810,-1.116) | **<0.001** |
| >=28.00 | -1.502 (-3.645,0.642) | 0.169 | -1.936 (-3.981,0.109) | 0.064 |
| **Residence** |  |  |  |  |
| Rural | REF |  | REF |  |
| Urban | 0.226 (-0.733,1.184) | 0.644 | -0.454 (-1.464,0.556) | 0.379 |
| Suburban | 1.776 (0.358,3.194) | 0.014 | 1.122 (-0.243,2.487) | 0.108 |
| **Ethnicity** |  |  |  |  |
| Han | REF |  |  |  |
| Ethnic minority | -0.535 (-1.597,0.527) | 0.323 |  |  |
| **Education** |  |  |  |  |
| Primary school or below | REF |  |  |  |
| Middle school/High school/Vocational school | 0.187 (-0.855,1.230) | 0.724 |  |  |
| Associate degree/Bachelor’s degree or above | 0.866 (-0.458,2.191) | 0.199 |  |  |
| **Monthly income per capita** |  |  |  |  |
| <5000 | REF |  | REF |  |
| 5000-10000 | 0.930 (-0.025,1.885) | 0.056 | 0.564 (-0.430,1.559) | 0.267 |
| >10000 | 1.232 (-0.232,2.696) | 0.099 | 0.422 (-1.156,2.001) | 0.600 |
| **Duration of cirrhosis** |  |  |  |  |
| 3 months or less | REF |  | REF |  |
| 4 months – 1 year | 1.121 (-0.274,2.515) | 0.115 | 0.507 (-0.850,1.864) | 0.464 |
| 1 year – 3 years | 1.574 (0.387,2.762) | 0.010 | 1.143 (-0.006,2.292) | 0.052 |
| More than 3 years | 1.946 (0.798,3.093) | 0.001 | 1.333 (0.204,2.462) | **0.021** |

All analyses used the full sample (N = 450).

**Table S4. Correlation analysis**

|  | **Knowledge** | **Attitude** | **Practice** |
| --- | --- | --- | --- |
| **Knowledge** | 1.000 |  |  |
| **Attitude** | 0.276 (P<0.001) | 1.000 |  |
| **Practice** | 0.278 (P<0.001) | 0.181 (P<0.001) | 1.000 |

All analyses used the full sample (N = 450).
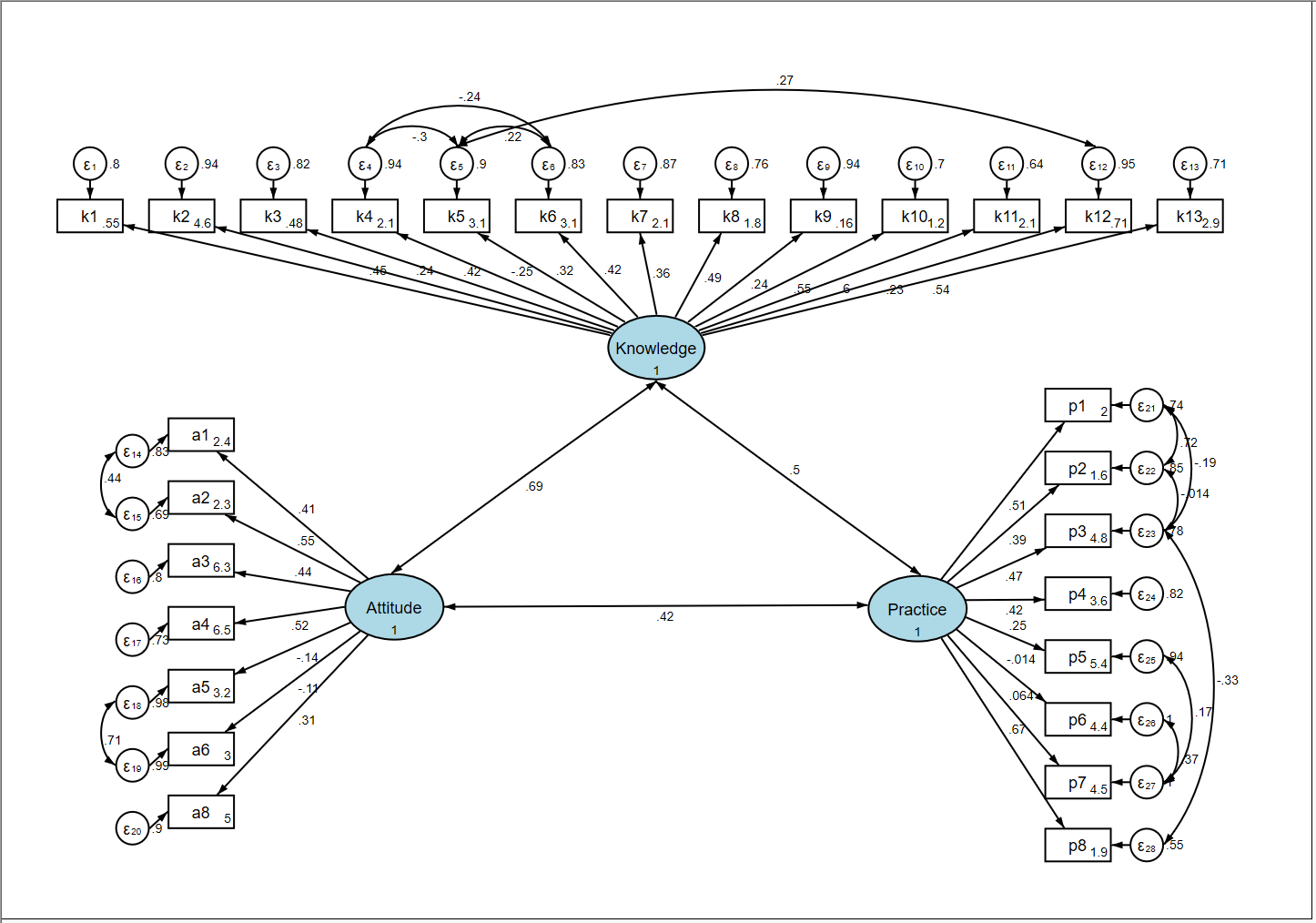


**Supplementary Figure 1. The confirmatory factor analysis**
