# Supplementary material for: Knowledge, attitudes, and practices of liver cirrhosis patients regarding dietary nutrition
Source: Front Nutr. 2026 Jan 6;12:1707256. doi: 10.3389/fnut.2025.1707256 (PMC12815832; doi:10.3389/fnut.2025.1707256)
Supplement: Supplementary file 2 [file Table_2.docx]

| Dear Participant,  We are researchers from xx *Hospital* and sincerely invite you to participate in our study. This study aims to understand the knowledge, attitudes, and practices of cirrhosis patients regarding nutrition and diet, providing a scientific basis for developing effective intervention strategies. These strategies may help more people improve their health in the future. Your participation in this study is voluntary. If you agree to participate, please review the following instructions:  1. Please complete the questionnaire. There are no right or wrong answers; simply answer based on your actual situation. If you encounter any questions during the process, feel free to ask us. After completion, please submit the questionnaire promptly.  2. This study is a simple questionnaire survey and will not cause any harm to your physical or mental health. However, it involves some personal information, such as your gender and age. We will strictly maintain confidentiality and ensure that your information is not disclosed, so please feel free to complete the questionnaire.  3. As a participant, you may inquire about study-related information and progress at any time. If you decide to withdraw from the study, please inform us, and your data will not be included in the study results.  Finally, we sincerely appreciate your valuable time and support for our scientific research!  □I acknowledge and agree that the collected data will be used for scientific research.  Informed Consent Signature:  Date of Participation: ____ Year ____ Month ____ Day |
| --- |

| **Part I-Basic Information** | |
| --- | --- |
| 1. **Your age** | Years old |
| **2. Your gender** | a. Male  b. Female |
| 1. **Your weight:** | kg |
| 1. **Your height:** | cm |
| **5. Your place of residence:** | a. Rural area b. Urban area c. Suburban area |
| **6. Your ethnicity:** | a. Han b. Ethnic minority If you belong to an ethnic minority, please specify: __________ |
| **7. Your education:** | a. Primary school or below b. Middle school/High school/Vocational school c. Associate degree/Bachelor’s degree d. Master’s degree or above |
| **8. In the past year, your household’s average monthly income per person (including in-kind income and rental income): ____ yuan** | a.<5000  b.5000-10000  c.10000-20000  d.>20000 |
| **9. How long have you had cirrhosis?** | Years |
| **10. What is the severity classification of your cirrhosis?** | a. A  b. B  c. C  d. Not sure |
| **11.1+2=4** | a. True b. False |

**Part II-Knowledge**

| **1. Have you ever learned about the "Dietary Guidelines for Chinese Residents," the "Dietary Pyramid for Chinese Residents," or other dietary nutrition recommendations?** | **a. Yes** | | | | **b. No** | |
| --- | --- | --- | --- | --- | --- | --- |
| **2. The six essential nutrients for the human body include carbohydrates, fats, proteins, minerals, vitamins, and water. To meet the body's needs, what foods should we eat?** | **a. Grains and tubers** | **b. Fish, poultry, eggs, lean meat, dairy products, beans, and bean products** | | **c. Vegetables and fruits** | | **d. Water** |
|  | **e. Oil** | **f. Sugar** | |  | |  |
| **3. For healthy individuals, ensuring a daily intake of at least ___ ml of water is beneficial for health. (Single choice with multiple options a–d)** | **a.1500** | | **b.1000** | | | **c.500** |
| **4. Malnutrition is one of the complications of cirrhosis. Which of the following factors make cirrhosis patients more prone to malnutrition?** | **a. Lack of appetite, unable to eat** | | **b. Poor digestion/absorption function** | | | **c. Increased body expenditure** |
|  | **d. Side effects of medications** | | **e. Decline in liver metabolism function** | | |  |
| **5. Malnutrition increases the risk of other complications in cirrhosis patients, including:** | **a. Edema** | | **b. Ascites** | | | **c. Infection** |
| **6. Signs of malnutrition in cirrhosis patients include:** | **a. Weakness** | | **b. Muscle loss** | | | **c. Weight loss** |
| **7. Cirrhosis can cause protein metabolism disorders. Increasing intake of high-quality proteins helps protect liver function. Which of the following foods are rich in high-quality protein?** | **a. Fish, poultry, lean meat, eggs, dairy products** | | **b. Soybeans and their products** | | | **c. Grains, tubers, and vegetables** |
| **8. Cirrhosis patients need nutritional screening and assessment. Are you aware of (or have you undergone) the following commonly used nutritional evaluation methods?** | **a. Measuring height, weight, and grip strength** | | **b. Nutritionist conducting body composition analysis** | | | **c. Blood tests to check albumin levels** |
|  | **d. Nutritionist asking dietary-related questions for evaluation** | |  | | |  |
| **9. Protein intake is very important for cirrhosis patients, but in the presence of the following complications, protein intake should be reduced or restricted.** | **a. Ascites** | | **b. Edema** | | | **c. Severe hepatic encephalopathy** |
| **10. In addition to protein, cirrhosis patients need to ensure the intake of the following nutrients:** | **a. Grains and tubers** | | **b. Fats** | | | **c. Vegetables and fruits** |
| **11. The following eating habits are suitable for cirrhosis patients:** | **a. Eating smaller meals more frequently** | | **b. Having a snack before bed** | | | **c. Binge eating** |
| **12. When ascites or edema occurs in cirrhosis, the intake of the following nutrients should be restricted:** | **a. Sodium (e.g., salt)** | | **b. Water** | | | **c. Sugar** |
| **13. The following are part of the nutritional support treatment for cirrhosis patients:** | **a. Antibiotic treatment** | | **b. Parenteral nutrition** | | | **c. Enteral nutrition** |

**Part-III Attitude**

**Please choose one of the options from "Strongly Agree" to "Strongly Disagree" according to your agreement with the statements.**

| 1. I am confident that I have a thorough understanding of the nutritional content of different foods. | **a. strongly agree** | **b. agree** | **c. neutral** | **d. disagree** | **e. strongly disagree** |
| --- | --- | --- | --- | --- | --- |
| 2. I am confident that I have a thorough understanding of the dietary needs for cirrhosis. | **a. strongly agree** | **b. agree** | **c. neutral** | **d. disagree** | **e. strongly disagree** |
| 3. I believe that ensuring an appropriate diet is very important for my disease recovery. | **a. strongly agree** | **b. agree** | **c. neutral** | **d. disagree** | **e. strongly disagree** |
| 4. I believe that dietary nutrition and medication treatment are equally important for my disease recovery. | **a. strongly agree** | **b. agree** | **c. neutral** | **d. disagree** | **e. strongly disagree** |
| 5. I believe that it is difficult to follow a strictly prescribed diet every day. | **a. strongly agree** | **b. agree** | **c. neutral** | **d. disagree** | **e. strongly disagree** |
| 6. I believe that it is difficult to ensure I eat on time every day. | **a. strongly agree** | **b. agree** | **c. neutral** | **d. disagree** | **e. strongly disagree** |
| 7. I believe that my disease sometimes affects my appetite. | **a. strongly agree** | **b. agree** | **c. neutral** | **d. disagree** | **e. strongly disagree** |
| 8. I believe that changing unhealthy habits like smoking and drinking alcohol can help with my disease recovery. | **a. strongly agree** | **b. agree** | **c. neutral** | **d. disagree** | **e. strongly disagree** |

**Part IV- Practice**

**Please select the option that most closely matches your situation.**

| 1. Have you participated in or are you currently participating in any education related to dietary and nutritional needs? | **a. Yes** | **b. No** |  |  |  |
| --- | --- | --- | --- | --- | --- |
| 2. Have you undergone a nutritional assessment? | **a. Yes** | **b. No** |  |  |  |
| 3. Do you follow the diet prescribed by your doctor at every meal? | **a. Always** | **b. Often** | **c. Sometimes** | **d. Rarely** | **e. Never** |
| 4. Are you able to follow the practice of eating smaller, more frequent meals in your daily diet? | **a. Always** | **b. Often** | **c. Sometimes** | **d. Rarely** | **e. Never** |
| 5. How often do you include carbohydrates such as rice or noodles in your daily diet? | **a. Always** | **b. Often** | **c. Sometimes** | **d. Rarely** | **e. Never** |
| 6. How often do you include protein-rich foods such as lean meat, soy products, dairy products, or fish in your daily diet? | **a. Always** | **b. Often** | **c. Sometimes** | **d. Rarely** | **e. Never** |
| 7. How often do you include vitamin- and fiber-rich foods such as vegetables and fruits in your daily diet? | **a. Always** | **b. Often** | **c. Sometimes** | **d. Rarely** | **e. Never** |
| 8. How often do you supplement your diet with amino acids, trace elements, vitamins, or other nutritional support through supplements or other means? | **a. Always** | **b. Often** | **c. Sometimes** | **d. Rarely** | **e. Never** |
